# MRI-derived radiomics for risk stratification of tumor deposits in rectal cancer: a dual-center study

**DOI:** 10.1186/s13244-025-02204-1

**Published:** 2026-02-02

**Authors:** Changjiang Zhang, Xiaojuan Deng, Zehong Cao, Feng Shi, Yi Yang, Yutong Chen, Huan Zhao, Xiaojing He, Xinjie Liu, Yindeng Luo

**Affiliations:** 1 Department of Radiology, The Second Affiliated Hospital of Chongqing Medical University, Chongqing, China; 2 Department of Radiology, The Third Affiliated Hospital of Chongqing Medical University, Chongqing, China; 3 Department of Research and Development, Shanghai United Imaging Intelligence, Shanghai, China; 4 Department of Radiology, Renji Hospital, School of Medicine, Chongqing University (The Fifth People’s Hospital of Chongqing), Chongqing, China

**Keywords:** Rectal cancer, Magnetic resonance imaging, Radiomics

## Abstract

**Objective:**

To evaluate the diagnostic utility of MRI-based radiomics in stratifying the risk of tumor deposits (TD) in patients with rectal cancer (RC).

**Materials and methods:**

This study retrospectively analyzed 729 patients with RC from two institutions (January 2018–August 2024). Patients were classified into three groups according to the number of TD: no TD (TD0), 1–2 TD (TD1-2), and ≥ 3 TD (TD3+). Radiomics features were extracted from the tumor and the largest nodule within the rectal mesentery on MRI images. Predictive models were developed with the XGBoost algorithm. Model performance was evaluated using the receiver operating characteristic curve, area under the curve, confusion matrix, precision, accuracy, recall, and F1 score.

**Results:**

Three hundred seventy-six patients were ultimately included and allocated into training, test, and validation sets. The tumor model (developed using tumor features) achieved AUCs of 0.871 (test set) and 0.848 (validation set), with corresponding accuracy, precision, recall, and F1 of 0.745/0.716, 0.764/0.688, 0.764/0.734, and 0.764/0.710, respectively. The nodule model (developed using the largest nodule) yielded AUCs of 0.839/0.804, accuracy of 0.673/0.637, precision of 0.571/0.614, recall of 0.800/0.686, and F1 of 0.667/0.648 in the test and validation sets, respectively. The fusion model, which combined tumor and nodule features, achieved enhanced performance with AUCs of 0.873/0.858, accuracy of 0.800/0.784, precision of 0.804/0.712, recall of 0.745/0.775, and F1 of 0.774/0.742, outperformed both individual models and two radiologists (accuracy 0.676/0.589).

**Conclusions:**

MRI-derived radiomics demonstrates significant potential for risk stratification of TD in RC.

**Critical relevance statement:**

The radiomics model integrating tumor features and maximal short-axis diameter of mesorectal nodules effectively predicts three distinct quantity-based categories of TD in RC, enabling preoperative risk stratification and assisting personalized treatment planning.

**Key Points:**

- Tumor and nodule features support effective stratification of TD.
- The fusion model for TD classification outperforms two radiologists.
- MRI-based radiomics aids TD risk stratification.

**Graphical Abstract:**

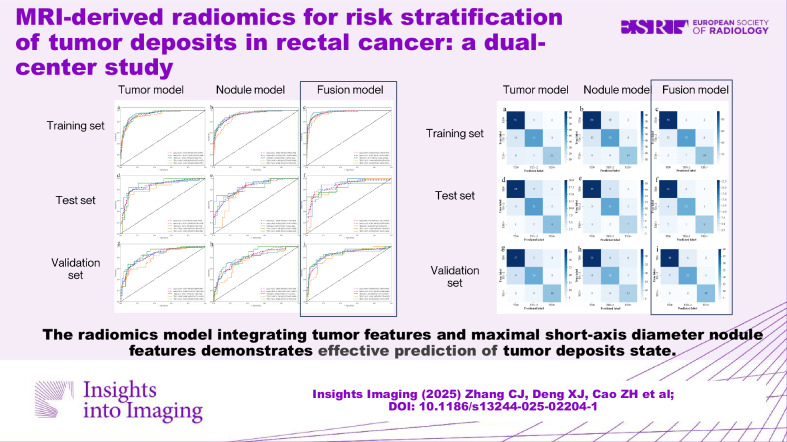

## Introduction

Colorectal cancer remains a significant global health challenge. In China, its incidence has risen sharply, making it the second most common malignancy, with rectal cancer (RC) being the most prevalent subtype [1, 2]. Tumor deposit (TD), defined as isolated tumor nodules lacking identifiable lymph node or vascular structures within the lymphatic drainage area of the primary tumor, are a key pathological feature of RC [3]. Extensive research has identified TD positivity as an independent prognostic factor in CRC. Reflecting this clinical relevance, the 8th edition of the AJCC cancer staging manual introduced a specific N1c category in the TNM staging system for patients with TD-positive lesions in the absence of lymph node metastasis [4].

However, growing clinical evidence has begun to challenge this binary classification approach. Recent studies suggest that quantifying the number of TD offers provides more accurate prognostic stratification than simply noting their presence or absence. Increasing TD counts have been consistently associated with worse outcomes, regardless of lymph node involvement. These findings underscore the limitations of current staging criteria and emphasize the biological significance of TD burden [5–7].

At present, clinical evaluation of TD relies predominantly on postoperative histopathological assessment, with no standardized protocols available for preoperative risk stratification based on TD quantification. MRI, recognized for its excellent soft tissue contrast, serves as the gold standard for preoperative locoregional staging and comprehensive risk evaluation in RC. Although morphology-based MRI criteria are currently the main imaging method for preoperative TD detection, this approach is limited by suboptimal diagnostic accuracy and significant variability between observers.

Radiomics, a quantitative imaging technique used to assess tumor microenvironment heterogeneity, has gained considerable attention for its ability to characterize the biological aggressiveness of RC. It has shown strong predictive performance for pathological risk factors, such as metastatic lymph node (MLN) involvement and extramural vascular invasion [8–10]. Critically, microenvironmental biomarkers manifest spatial distribution patterns transcending tumor margins into adjacent peritumoral anatomical compartments. Several previous studies have highlighted that advanced radiomic analysis of mesorectal nodule structures, particularly through detailed quantification of nodule features, can significantly improve the predictive capacity for TD assessment [10, 11]. This approach addresses limitations of earlier radiomics techniques by expanding feature extraction beyond the primary tumor area. We hypothesized that radiomic features derived from MRI, specifically extracted from both the primary tumor and the largest mesorectal nodule, hold significant discriminative potential for stratifying TD burden in RC. These distinct anatomical regions may capture complementary microenvironmental and morphological characteristics associated with TD development and progression.

Accordingly, by integrating features from both the tumor and adjacent mesorectal nodules, we aimed to enhance accuracy in preoperative evaluation of TD status.

## Materials and methods

### Study population

The study protocol was approved by the Ethics Committees of the Second Affiliated Hospital (approval number: 2024-79) and the Third Affiliated Hospital of Chongqing Medical University (approval number: 2024-66), with a waiver of informed consent. A total of 729 patients who underwent radical resection for RC between January 2018 and August 2024 at two institutions (designated A and B) were initially screened. Inclusion criteria were: (1) histologically confirmed rectal adenocarcinoma with documented TD counts, (2) no prior anticancer therapy, and (3) preoperative MRI performed within two weeks before surgery. Exclusion criteria were: (1) invisible tumor on T2-weighted imaging (T2WI) (*n* = 32), (2) no nodules with short-axis diameter (SD) ≥ 3 mm (*n* = 156), (3) poor image quality (*n* = 59), (4) missing TD status (*n* = 68), and (5) prior malignancy (*n* = 38)

**Fig. 1:**
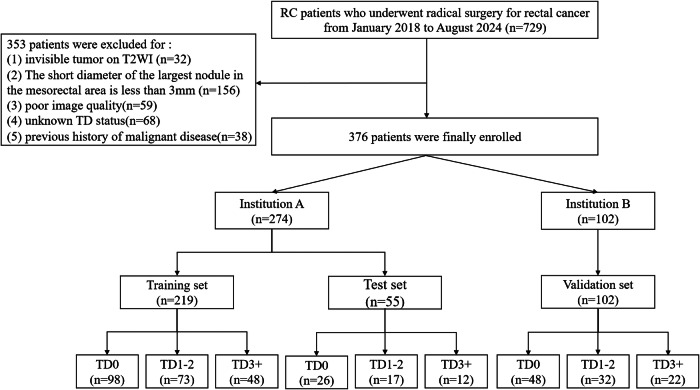
The workflow for patient enrollment and assignment

A total of 376 patients were ultimately included. Based on TD counts from histopathology, patients were grouped into TD0 (no TD), TD1–2 (1–2 TDs), and TD3+ (≥ 3 TDs). Patients from Institution A were randomly split into training and internal test sets (8:2 ratio), while patients from Institution B served as the external validation cohort. The clinical and pathologic features for all enrolled patients were retrospectively obtained from the institutional medical records. The inclusion process is shown in Fig. 1.

### Histopathological analysis

Two gastrointestinal pathologists (> 8 years of experience) assessed TD status on hematoxylin and eosin (H&E)-stained slides. TDs were defined as irregular, discrete carcinoma nodules in the pericolorectal adipose tissue lacking residual lymphoid architecture typical of lymph nodes.

### Image acquisition and preprocessing

Institution A used a 3.0-T MRI scanner (Ingenia CX, Philips Healthcare) and a 1.5-T scanner (Avanto Eco, Siemens Healthineers), while Institution B used a 3.0-T MRI scanner (Discovery 750W, GE Healthcare). At both institutions, non-fat-suppressed T2WI was performed in the oblique axial plane, perpendicular to the involved rectal segment. Detailed MRI acquisition parameters are detailed in Table 1.

**Table 1:** MRI acquisition parameters

	Institution A		Institution B
	PHILIPS	SIEMENS	GE
	Ingenia CX	Avanto eco	Discovery 750w
Field strength (T)	3.0	1.5	3.0
Fat suppression	Non	Non	Non
Repeat time (ms)	1541	1500	4046
Echo time (ms)	157	97	70
Number of average	2	2	2
Echo train length	130	60	16
Slice thickness (mm)	0.9	2	3
Slice gap (mm)	0	0	0.5

All T2WI images underwent N4 bias field correction and were resampled to a voxel size of 1 mm × 1 mm × 1 mm

### Image analysis

Two radiologists (with 3 and 5 years of gastrointestinal imaging experience, respectively) independently segmented regions of interest (ROIs) on T2WI using ITK-SNAP (v3.6.1, http://www.itksnap.org). They delineated the primary tumor and the largest SD mesorectal nodule, blinded to histopathology and guided by multiparametric sequences. Inter-observer reproducibility was evaluated on a random subset of 100 training cases.

**Fig. 2:**
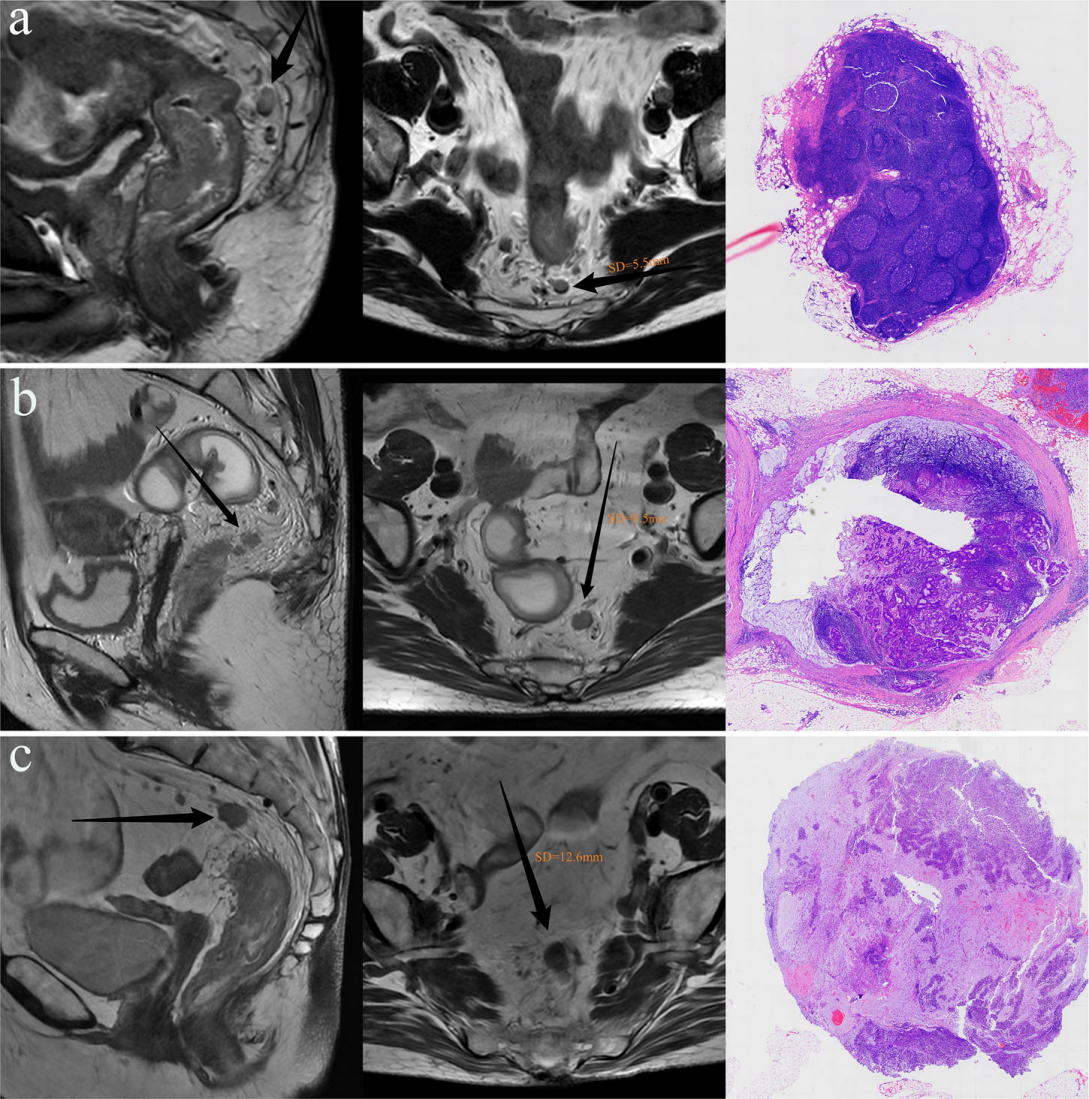
Representative MRI and corresponding histopathological images from patients with different TD statuses. **a** A 75-year-old male with the largest nodule measuring 5.5 mm in SD on T2WI. Histopathological examination identified no TD; the largest nodule was confirmed as a normal lymph node. **b** A 55-year-old female with the largest nodule measuring 8.5 mm in SD on T2WI. Histology revealed two TDs, and the largest nodule was an MLN. **c** A 66-year-old female with the largest nodule measuring 12.6 mm in SD on T2WI. Histology identified five TDs, and the largest nodule was pathologically confirmed as a TD

Additionally, to assess model performance, Radiologists A and B received training from a senior radiologist (> 10 years of experience) and independently reviewed preoperative MRI from the validation set. They categorized TD status (TD0, TD1–2, or TD3+) based on established MR–TD morphological criteria, defined as an irregular nodule within or along a venous channel, continuous with major mesorectal venous branches but distinct from the primary tumor [12, 13]. As shown in Fig. 2.

### Radiomics feature extraction and selection

A total of 1743 radiomics features were extracted from each ROI (tumor and nodule) using Pyradiomics (v3.1.0, https://pypi.org/project/pyradiomics). The features were categorized as follows: first-order statistics (*n* = 342), textural features (*n* = 1387) including gray-level co-occurrence matrix (GLCM), gray-level dependence matrix (GLDM), gray-level run-length matrix (GLRLM), gray-level size zone matrix (GLSZM), and neighboring gray-tone difference matrix (NGTDM), and 3D morphological features (*n* = 14). A pre-fusion strategy combined features from both regions. All features were standardized using *Z*-score normalization before modeling.

Each feature set (tumor, nodule, and fusion) underwent an identical preprocessing pipeline. Feature selection comprised three sequential steps: (1) initial filtration using two-tailed t-tests (*p* < 0.05) and inter-observer coherence index (ICC ≥ 0.75); (2) elimination of redundant features via Pearson correlation (|*r*| ≥ 0.9); and (3) final selection with least absolute shrinkage and selection operator regression using ten-fold cross-validation to optimize the feature subset.

### Radiomics modeling and evaluation

To address class imbalance among TD subgroups, the synthetic minority over-sampling technique (SMOTE) was applied during training to augment the TD1–2 and TD3+ groups. Three models—tumor, nodule, and fusion—were subsequently developed using the extreme gradient boosting (XGBoost) algorithm, with hyperparameters optimized through five-fold cross-validation.

**Fig. 3:**
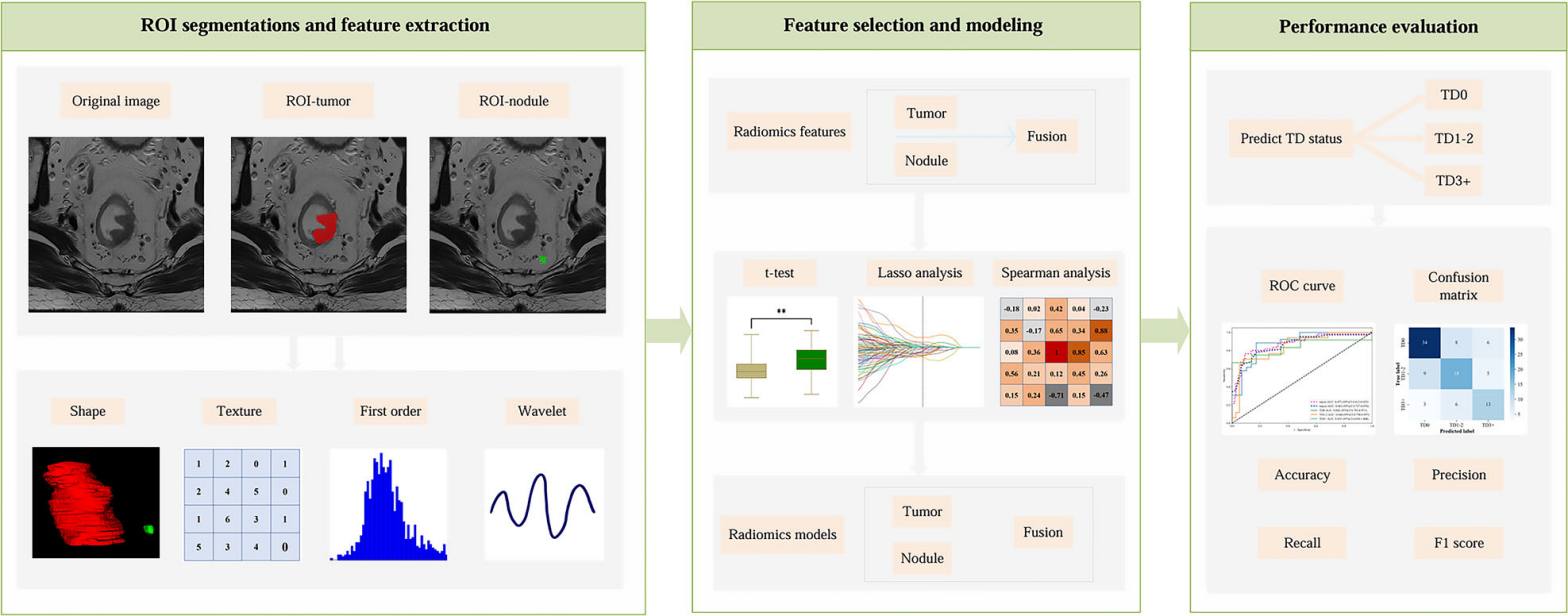
The radiomics workflow of this study

Model performance was evaluated within a multidimensional framework. Receiver operating characteristic (ROC) curves were generated, and key metrics—including area under the curve (AUC), precision, accuracy, recall, and F1-score—were derived from confusion matrices using a micro-averaging approach to mitigate class imbalance. The overall workflow is illustrated in Fig. 3.

### Statistical analysis

Statistical analyses were conducted using Python (v3.12.1, https://www.python.org) and SPSS 26.0 (IBM) software. Continuous variables were assessed for normality using the Kolmogorov–Smirnov test and expressed as mean ± standard deviation or median (interquartile range), as appropriate. Group comparisons were performed using analysis of variance (ANOVA) for continuous variables and the Pearson chi-square test for categorical variables. A two-tailed *p* < 0.05 was considered statistically significant.

## Results

### Patients’ characteristics

This study included 376 patients (211 males and 165 females) from two institutions (Institution A: *n* = 274; Institution B: *n* = 102). Based on TD count, patients were categorized into TD0 (*n* = 172), TD1–2 (*n* = 124), and TD3+ (*n* = 82) groups. The mean ages in the training, test, and validation sets were 64 ± 12, 60 ± 14, and 61 ± 14 years, respectively. Among baseline characteristics, only the T stage differed significantly across the three datasets (*p* = 0.010) (Table 2).

**Table 2:** Baseline characteristics of the study population

Characteristics	Training set (*n* = 219)	Test set (*n* = 55)	Validation set (*n* = 102)	*p* value
Age (years)	64 ± 12	60 ± 14	61 ± 14	0.069
Gender				0.743
Male	125 (55%)	32 (58%)	54 (53%)	
Female	94 (45%)	23 (42%)	48 (48%)	
Tumor location				0.715
Low	58 (26%)	18 (33%)	34 (33%)	
Middle	120 (55%)	27 (49%)	49 (48%)	
High	41 (19%)	10 (18%)	19 (19%)	
SD of the largest nodules (mm)				0.769
	5.9 (4.7, 8.3)	6.3 ± 2.4	6.3 (4.6, 7.7)	
T stage				0.010
T1–T2	31 (14%)	15 (27%)	27 (26%)	
T3–T4	188 (86%)	40 (73%)	75 (74%)	
N stage				0.410
N0	44 (20%)	11 (20%)	27 (26%)	
N1–N2	175 (80%)	44 (80%)	75 (74%)	
EMVI status				0.716
Negative	103 (47%)	25 (46%)	43 (42%)	
Positive	116 (53%)	30 (54%)	59 (58%)	
TDs status				
TD0	98 (47%)	26 (47%)	48 (47%)	
TD1–2	75 (31%)	17 (31%)	32 (31%)	
TD3+	48 (22%)	12 (22%)	22 (22%)	

Tumor location: low (< 5 cm from the anal verge), middle (5–10 cm), high (≥ 10 cm)

*SD* short-axis diameter, *TD* tumor deposit, *EMVI* extramural vascular invasion

### Radiomic modeling features

Following feature selection, three optimized radiomics feature sets were obtained (Table S1). The tumor, nodule, and fusion models incorporated 20, 16, and 21 features, respectively. The tumor model included 5 first-order, 5 GLCM, 1 GLDM, 3 GLRLM, 3 GLSZM, 2 NGTDM, and 1 shape feature. The nodule model comprised 1 first-order, 1 GLCM, 2 GLDM, 3 GLRLM, 6 GLSZM, 2 NGTDM, and 1 shape feature. The fusion model contained 3 first-order, 6 GLCM, 2 GLDM, 8 GLSZM, and 2 NGTDM features.

### Performance of radiomics models

Tables 3 and 4 summarize the diagnostic performance of the three radiomics models, evaluated on the training, testing, and validation cohorts, respectively. Of these, the fusion model outperformed the tumor and nodule models across most metrics in the training set. It achieved the highest accuracy (0.872 vs 0.775 and 0.670), AUC (0.961 vs 0.930 and 0.926), precision (0.854 vs 0.808 and 0.776), and F1-score (0.861 vs 0.816 and 0.771). Although its recall (0.849) was slightly lower than that of the nodule model (0.909), it was comparable to the tumor model (0.863), indicating strong overall performance.

**Table 3:** Overall performance of different models based on the training set

Models	AUC (95% CI)	Accuracy	Precision	Recall	F1
Tumor model	0.930 (0.911–0.950)	0.808	0.775	0.863	0.816
Nodule model	0.926 (0.906–0.946)	0.776	0.670	0.909	0.771
Fusion model	0.961 (0.947–0.975)	0.854	0.872	0.849	0.861

*AUC* the area under the receiver operating characteristic curve, *CI* confidence interval

**Table 4:** Overall performance of different models based on test and validation sets

Models		Tumor model			Nodule model			Fusion model		
		Precision	Recall	F1	Precision	Recall	F1	Precision	Recall	F1
Test set (*n* = 55)										
TD0		0.833	0.769	0.800	0.944	0.654	0.723	0.815	0.846	0.830
TD1–2		0.667	0.706	0.686	0.500	0.824	0.622	0.786	0.647	0.710
TD3+		0.692	0.750	0.720	0.429	0.750	0.545	1.000	0.583	0.737
Micro		0.764	0.764	0.764	0.571	0.800	0.667	0.804	0.745	0.774
AUC (95% CI)		0.871 (0.811–0.931)			0.839 (0.775–0.902)			0.873 (0.812–0.933)		
Accuracy		0.745			0.673			0.800		
Validation set (*n* = 102)										
TD0		0.755	0.833	0.792	0.786	0.688	0.733	0.854	0.729	0.787
TD1–2		0.704	0.594	0.644	0.538	0.656	0.592	0.700	0.656	0.677
TD3+		0.429	0.955	0.592	0.353	0.818	0.493	0.529	0.818	0.643
Micro		0.688	0.735	0.710	0.614	0.686	0.648	0.712	0.775	0.742
AUC (95% CI)		0.848 (0.803–0.893)			0.804 (0.753–0.856)			0.858 (0.809–0.907)		
Accuracy		0.716			0.637			0.784		

**Fig. 4:**
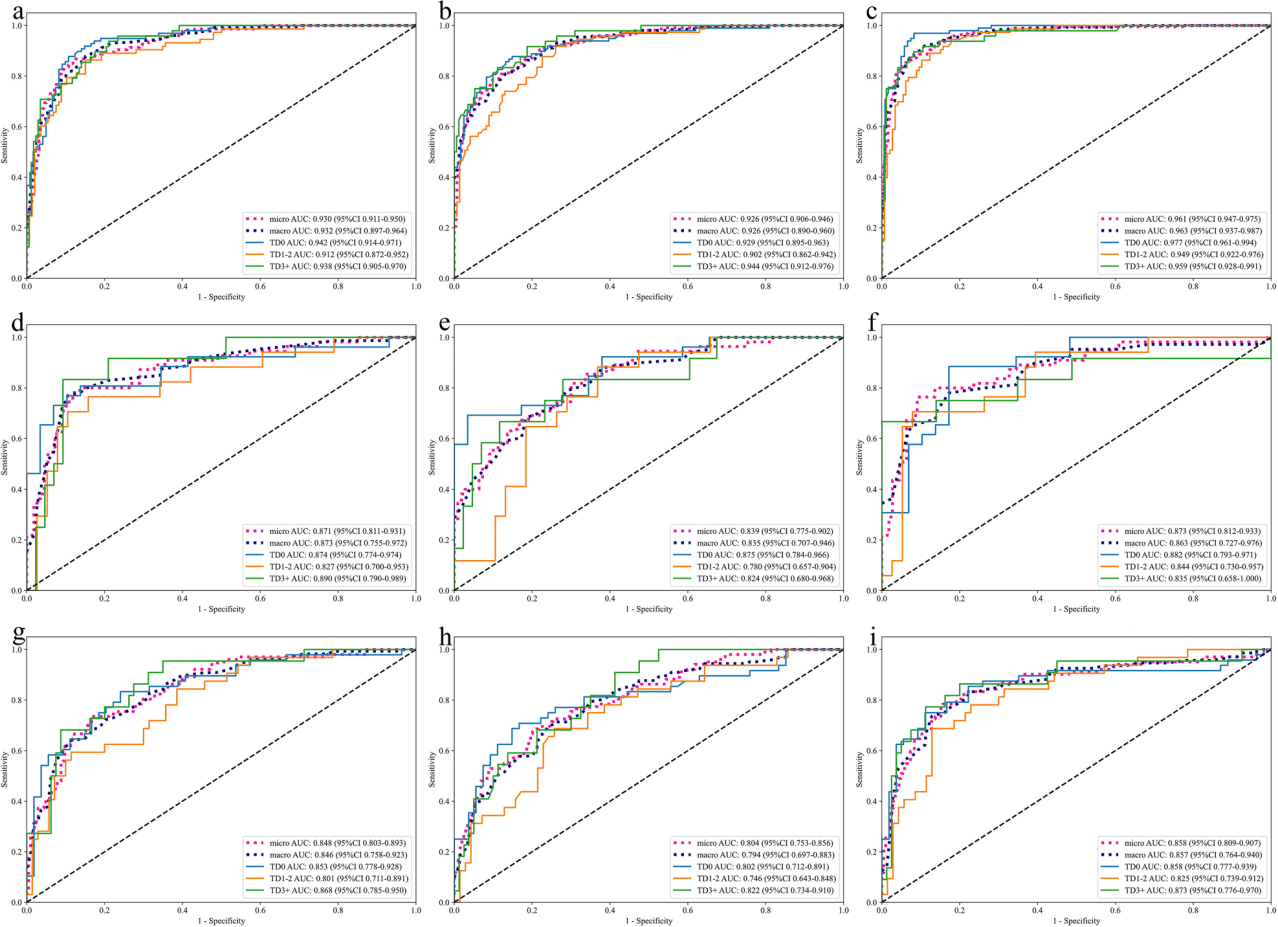
ROC curves for different models on training, test, and validation sets. **a**, **d**, **g** Show tumor model ROC curves for training, internal test, and external validation sets. **b**, **e**, **h** Display nodule model results across corresponding datasets. **c**, **f**, **i** Present fusion model performance in these three phases

**Fig. 5:**
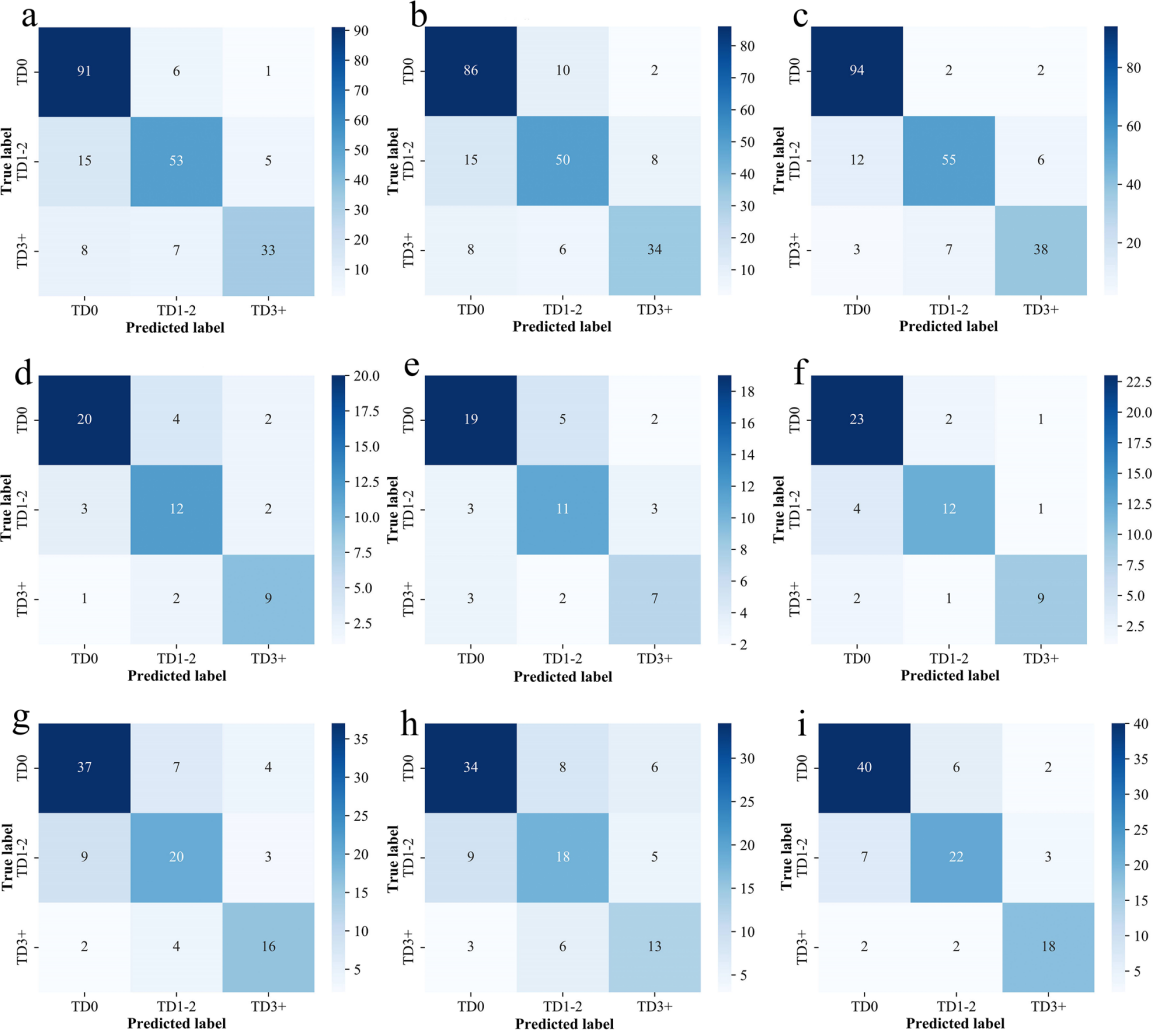
Confusion matrix for different models on training, test, and validation sets. **a**, **d**, **g** Show tumor confusion matrix for training, internal test, and external validation sets. **b**, **e**, **h** display nodule model results across corresponding datasets. **c**, **f**, **i** Present fusion model performance in these three phases

Consistent trends were observed in the internal test and external validation sets, where the fusion model achieved superior accuracy (test: 0.800; validation: 0.784) and AUC (test: 0.873; validation: 0.858), with balanced precision, recall, and F1-scores. In contrast, the tumor (accuracy: 0.745/0.716; AUC: 0.871/0.848) and nodule (accuracy: 0.673/0.637; AUC: 0.839/0.804) models showed lower diagnostic performance. The ROC curves and confusion matrices across the three datasets demonstrated that the fusion model achieved the best overall classification performance, exhibiting the highest prediction accuracy and AUC values for TD0 through TD3+ (Figs. 4 and 5). In subgroup classification, the fusion model achieved the highest F1-scores in the test set for TD0 (0.830), TD1–2 (0.710), and TD3+ (0.734). In the validation set, it maintained comparable results, although slightly underperformed the tumor model for TD0 classification (F1-score: 0.787 vs 0.792). The fusion model preserved strong validation performance for TD1–2 (F1 = 0.677) and TD3+ (F1 = 0.643) groups. Despite reduced performance across all subgroups in the validation set relative to training, it consistently achieved higher AUCs than the other models, underscoring its superior discriminative capability.

### Comparison of the best radiomics model and radiologists

In the external validation set, the fusion model demonstrated superior classification performance compared with both radiologists (Table 5). It achieved higher accuracy (0.783) than Radiologist A (0.589) and Radiologist B (0.676). The model also exhibited greater sensitivity across all subgroups (TD0: 0.729 vs A/B: 0.708/0.771; TD1–2: 0.626 vs 0.500/0.625; TD3+: 0.818 vs 0.455/0.545). In addition, it achieved higher specificity for TD0 (0.889) and TD1–2 (0.871) compared with both radiologists, although its specificity for TD3+ (0.800) was slightly lower than that of Radiologist A (0.825) and Radiologist B (0.900).

**Table 5:** Performance of radiologists and radiomics model on validation set (*n* = 102)

	Fusion model		Radiologist A		Radiologist B	
	Sensitivity	Specificity	Sensitivity	Specificity	Sensitivity	Specificity
TD0	0.729	0.889	0.708	0.815	0.771	0.833
TD1-2	0.626	0.871	0.500	0.743	0.625	0.814
TD3+	0.818	0.800	0.455	0.825	0.545	0.900
Accuracy	0.784		0.589		0.676	

## Discussion

Clinical studies have consistently shown that a higher TD burden is strongly associated with poorer clinical outcomes, with increasing TD counts correlating with progressively lower postoperative survival rates [14–17]. In this study, we evaluated the diagnostic value of radiomic features extracted from the primary tumor on T2WI and the largest mesorectal nodule (based on maximum SD) for preoperative TD risk stratification. The fusion model integrating features from both regions achieved superior classification performance compared with single-region models, with accuracies of 0.800 (44/55 cases) in the test set and 0.784 (80/102 cases) in the validation set. Moreover, it outperformed two radiologists, demonstrating accuracy improvements of 0.195 and 0.108, respectively. These results highlight the effectiveness of the dual-region radiomics model in preoperatively stratifying TD risk in RC patients and its potential to enhance clinical decision-making and treatment planning.

Current preoperative TD assessment in RC primarily relies on MRI-based morphological evaluation [12, 18]. This conventional approach depends on subjective image interpretation, resulting in high interobserver variability and limited diagnostic consistency. These limitations underscore the need for objective, standardized, and reproducible tools to improve the accuracy and reliability of TD risk stratification in RC management.

Previous studies on radiomics for preoperative TD assessment have mainly focused on binary patient-level classification. Atre et al [19] analyzed texture features from 2D-ROI on T2WI of the largest TD and MLN, identifying skewness as a key differentiator (AUC = 0.70, sensitivity = 70%, specificity = 72%). Yang et al [20] developed a T2WI-based model using tumor dimension features, achieving improved performance (AUC = 0.80, sensitivity = 72%, specificity = 94%). Zhang et al [10] further advanced this approach by employing contrast-enhanced CT arterial-phase images with 3D-ROI from both the primary tumor and perirectal nodules. Their nodule-based nomogram outperformed tumor-based models, demonstrating higher diagnostic accuracy (AUC = 0.918, sensitivity = 88.3%, specificity = 82.6%) and underscoring the predictive value of features derived from peritumoral nodules.

In this study, a combined feature extraction strategy incorporating both tumor and maximal nodule regions was applied to explore multiregional radiomics characteristics on T2WI for three-category TD classification. Through a rigorous feature selection process, three optimized models were developed: the tumor model included 20 features, the nodule model comprised 16, and the fusion model integrated 21 features across both regions (11 from the tumor and 10 from the nodule). The three models exhibited different predictive performance levels, reflecting the distinct contributions of tumor- and nodule-based features. The nodule model demonstrated the lowest overall classification performance, which is likely attributed to the fact that not all the largest mesorectal nodules visible on T2WI corresponded directly to TD in this study. Some represented metastatic or benign enlarged lymph nodes, which reduced model specificity. However, the primary objective of this study was to stratify the overall TD-related risk in RC rather than determine the pathological nature of individual nodules. Notably, a significant difference was observed in the maximum SD of mesorectal nodules across TD groups: TD0 = 5.2 (4.0, 6.3) mm, TD1–2 = 6.4 (5.2, 7.7) mm, and TD3 + = 8.7 ± 3.0 mm (*p* < 0.001), suggesting that an increase in the SD of the largest mesorectal nodule is associated with elevated TD risk. Selecting the largest nodule for analysis improved both stratification accuracy and reproducibility. Importantly, the superior performance of the fusion model likely stems from both the primary tumor and the largest mesorectal nodule, supporting the hypothesis that tumor microenvironmental remodeling extending from the primary tumor lesion into adjacent perirectal tissues contributes to TD formation [21, 22].

Additionally, we compared the diagnostic performance of the fusion model with that of radiologists, and the fusion model demonstrated clear superiority (accuracy: 0.784 vs 0.589/0.676). This highlights its potential to reduce diagnostic subjectivity, an important advancement given the prognostic significance of TD count in the clinical staging of RC [23–25]. When the SD of the largest mesorectal nodule is ≥ 3 mm, the fusion model significantly improves the accuracy of preoperative TD risk assessment, thereby facilitating a more precise prognostic evaluation and potential clinical decision optimization. For instance, patients in the TD3+ group could benefit from intensified neoadjuvant therapy or wider surgical margins, while those in the TD0 group, lacking additional risk factors, may avoid unnecessary overtreatment [26].

This study has several limitations. Firstly, because this was a retrospective study, selection bias may have been introduced. Secondly, due to the lack of a direct correlation between the images and pathological sections, the features of the largest nodule do not necessarily reflect the true TD characteristics. Thirdly, due to the short follow-up period for most patients, the prognostic value of the radiomics model could not be evaluated. Lastly, the model’s predictions should be interpreted alongside clinical information, as a supplement to radiologists’ interpretations. Future studies with larger samples, advanced imaging technologies, and deep learning will improve its accuracy and applicability.

## Conclusion

Our study demonstrates that a T2WI-based radiomics model effectively preoperatively stratifies TD risk in RC. By integrating features from both the primary tumor and the largest mesorectal nodule via the XGBoost algorithm, the fusion model captures complementary tumor microenvironment information and achieves superior predictive performance over single-region models.

## Supplementary information

ELECTRONIC SUPPLEMENTARY MATERIAL

## Data Availability

All data supporting the findings of this study are available from the corresponding author upon reasonable request. The data are not publicly available due to privacy/ethical restrictions, but will be provided to qualified researchers subject to compliance with institutional requirements.

## Abbreviations

AUC: Areas under the curve
CI: Confidence interval
RC: Rectal cancer
ROC: Receiver operating characteristic
ROIs: Regions of interest
SD: Short-axis diameter
T2WI: T2-weighted imaging
TD: Tumor deposit
XGboost: Extreme gradient boosting tree
