# Supplementary material for: MRI-derived radiomics for risk stratification of tumor deposits in rectal cancer: a dual-center study
Source: Insights Imaging. 2026 Feb 2;17:31. doi: 10.1186/s13244-025-02204-1 (PMC12864629; doi:10.1186/s13244-025-02204-1)
Supplement: Supplementary file 1 — ELECTRONIC SUPPLEMENTARY MATERIAL [file 13244_2025_2204_MOESM1_ESM.pdf]

MRI-derived radiomics for risk stratification of tumor deposits in rectal cancer: a dual-center study

ELECTRONIC SUPPLEMENTARY MATERIAL

Table S1 The final features for three radiomics models.

Model	Feature name	Importance
Tumor model	Tumor_isquareroot_glrlm_LongRunEmphasis	0.08754679
	Tumor_ilbp_3D_k_glrlm_ShortRunEmphasis	0.08514089
	Tumor_ilbp_3D_k_firstorder_Range	0.07593433
	Tumor_iwavelet_HLL_firstorder_10Percentile	0.07587277
	Tumor_ilbp_3D_k_glszm_ZonePercentage	0.057160072
	Tumor_isquare_glcm_Idn	0.056611
	Tumor_isquareroot_glszm_SizeZoneNonUniformity	0.05346129
	Tumor_iwavelet_LLL_firstorder_Kurtosis	0.05129515
	Tumor_ioriginal_shape_Elongation	0.04972889
	Tumor_iwavelet_LLH_glcm_Idn	0.049247768
	Tumor_ilogarithm_ngtdm_Strength	0.048282932
	Tumor_ilog_sigma_1_0_mm_3D_glcm_Correlation	0.04502835
	Tumor_iwavelet_LHL_ngtdm_Complexity	0.041851796
	Tumor_iexponential_glcm_Correlation	0.038142666
	Tumor_ilog_sigma_3_0_mm_3D_firstorder_Skewness	0.036093567
	Tumor_iwavelet_LLH_glcm_Imc2	0.03466158
	Tumor_ilog_sigma_3_0_mm_3D_gldm_SmallDependenceLowGrayLevelEmphasis	0.032908686
	Tumor_ilbp_3D_m2_glszm_SmallAreaLowGrayLevelEmphasis	0.030659456
	Tumor_isquareroot_firstorder_Minimum	0.02760953
	Tumor_iexponential_glrlm_RunLengthNonUniformity	0.022762414
Nodule model	Nodule_ilbp_3D_k_glrlm_LongRunLowGrayLevelEmphasis	0.14392616
	Nodule_isquareroot_glrlm_LongRunEmphasis	0.12458229
	Nodule_ilbp_3D_m1_glszm_ZoneEntropy	0.09293728
	Nodule_ilogarithm_glszm_ZonePercentage	0.07988202
	Nodule_ilog_sigma_3_0_mm_3D_ngtdm_Complexity	0.06535221
	Nodule_isquare_glszm_SmallAreaLowGrayLevelEmphasis	0.06509788
	Nodule_iwavelet_LLH_ngtdm_Strength	0.05881771
	Nodule_iwavelet_HLH_gldm_LargeDependenceLowGrayLevelEmphasis	0.05881771
	Nodule_iwavelet_HLL_glszm_LargeAreaLowGrayLevelEmphasis	0.04740017

	Nodule_iwavelet_HHL_firstorder_Median	0.047297016
	Nodule_ioriginal_shape_Maximum2DDiameterColumn	0.046902653
	Nodule_iwavelet_LLL_glcm_ClusterShade	0.046783518
	Nodule_ilbp_3D_k_glszm_SizeZoneNonUniformity	0.045246247
	Nodule_ilogarithm_glszm_LargeAreaHighGrayLevelEmphasis	0.02985051
	Nodule_iexponential_glrlm_ShortRunHighGrayLevelEmphasis	0.028980779
	Nodule_iexponential_gldm_DependenceVariance	0.02723568
Fusion model	Nodule_ilogarithm_glszm_GrayLevelNonUniformityNormalized	0.120069355
	Nodule_iwavelet_LLL_glszm_SizeZoneNonUniformity	0.09019681
	Nodule_iwavelet_LLH_ngtdm_Strength	0.074101575
	Tumor_ilog_sigma_1_0_mm_3D_glcm_Imc1	0.07023497
	Nodule_ilog_sigma_3_0_mm_3D_firstorder_RootMeanSquared	0.061094094
	Tumor_ilbp_3D_k_glszm_ZonePercentage	0.05498822
	Tumor_iwavelet_LLL_glszm_LargeAreaHighGrayLevelEmphasis	0.05480658
	Tumor_isquareroot_glszm_SizeZoneNonUniformity	0.052969232
	Nodule_iexponential_gldm_DependenceVariance	0.05102633
	Tumor_isquare_ngtdm_Contrast	0.049957585
	Tumor_iexponential_glcm_Correlation	0.04630552
	Nodule_isquare_glszm_SmallAreaLowGrayLevelEmphasis	0.041821003
	Tumor_ilbp_3D_k_firstorder_Range	0.04016911
	Tumor_iwavelet_HLL_glcm_MaximumProbability	0.039362624
	Tumor_ilog_sigma_1_0_mm_3D_firstorder_Skewness	0.027937086
	Nodule_iwavelet_LLL_glcm_ClusterShade	0.027371785
	Tumor_ilbp_3D_m2_glcm_Correlation	0.022302132
	Nodule_ilbp_3D_m1_glszm_ZoneEntropy	0.02045238
	Nodule_isquare_gldm_DependenceNonUniformityNormalized	0.019379238
	Nodule_ilbp_3D_m2_glszm_SmallAreaEmphasis	0.01919759
	Tumor_iwavelet_LLH_glcm_Imc2	0.01625682
